# CHLA 2026 CONFERENCE LIGHTNING TALKS / ABSC CONGRÈS 2026 PRÉSENTATIONS ÉCLAIR

**DOI:** 10.29173/jchla29967

**Published:** 2026-08-03

Robin Parker, Jackie Phinney, Louise Gillis, Shelley McKibbon, Melissa Rothfus

Dalhousie University

**Introduction:** For over two decades, our academic health library has supported knowledge synthesis (KS) projects through individual and group instruction, a dedicated LibGuide, webinars, asynchronous online tutorials, mediated search services, and co-authorship. Like many academic libraries, we’ve seen rising demand across all levels of training and collaboration, requiring increased time and resources. However, communicating the value and impact of KS work to the broader library system and university administration remains a challenge. **Description:** To address this gap, we developed a holistic assessment plan for KS support, informed by the CARL Library Impact Framework and evaluation models from other institutions. Using a logic model approach, the plan incorporates both quantitative and qualitative data to evaluate inputs, outputs, outcomes, and long-term impact so we can illustrate the interconnected work within and beyond the library to achieve teaching and research objectives. **Outcomes:** The resulting data will help document the evolution of KS support and highlight our library’s contributions across institutional research, academic, and service priorities. This framework may also guide other academic health libraries facing similar challenges balancing demand with limited resources. **Discussion:** In times of technological change and constrained budgets, it is more important than ever to align the work done in libraries with institutional strategic goals. Clearly articulating the impact of KS support on research and academic programming strengthens the case for maintaining or increasing investment. By demonstrating value, libraries can advocate for the resources needed to meet growing demand and ensure the continued sustainability of KS work in academic health libraries.

