# Supplementary material for: CHLA 2026 CONFERENCE LIGHTNING TALKS / ABSC CONGRÈS 2026 PRÉSENTATIONS ÉCLAIR
Source: J Can Health Libr Assoc. 2026 Aug 3;47(2):79–87. doi: 10.29173/jchla29967 (PMC13590142; doi:10.29173/jchla29967)
Supplement: Related texts

# Abstract: LT2. ''Not so solo'' anymore! The power of informal peer support

Jessie Cunningham^1^, Lisa Shaver^2^, Celeste Ramnarine^3^, Amanda Caputo^4^

^1^The Hospital for Sick Children, ^2^The Children's Hospital of Eastern Ontario, ^3^DistillerSR Inc., ^4^The Sault Area Hospital

Many library professionals that work alone, can sometimes feel like they work in a bubble. It’s often difficult to readily source peer support and feedback. Additionally, it’s difficult not having access to fresh perspectives, particularly when trying to build and maintain professional skills and competencies. Professional associations such as CHLA-ABSC do offer opportunities for support, community, and development, but often these fora can be quite formal, and are not necessarily appropriate places for members to speak frankly, and with a sense of psychological safety. This highlights the need for informal connections to fill in those gaps. By reaching out informally to peers in external organizations, Information professionals can provide neutral, judgement-free support that might not otherwise be available. In 2024, librarians from the Children's Hospital of Eastern Ontario (CHEO), The Hospital for Sick Children (SickKids), Oak Valley Health (Markham Stouffville Hospital), and the Sault Area Hospital, connected to create an informal virtual space, to obtain peer support for common issues, resolving tricky literature searches, PRESS reviewers if needed, and a safe place to vent. It’s called the “Not-So-Solo-Librarians” (NSSL) group. It's an informal network of solo medical librarians that have harnessed the power of informal peer support; allowing for flexibility, psychological safety, and a reliable sounding board. These opportunities can also enable “career reflections”, allowing members to explore how to make the most of their own career paths. We encourage other similarly situated health information professionals, to form their own informal networks, to enable collegial and mutually beneficial relationships.

# Abstract: LT3. Navigating change: transitioning towards a team-based model

Carly Schanock, Casey Phillips, Melissa Funaro, Alexandria Brackett, Holly Grossetta Nardini, Zsuzsanna Nemeth

Whitney Medical Library - Yale University

The Clinical Research and Education Librarians at the Harvey Cushing/John Hay Whitney Medical Library (CWML) are piloting a transition from a liaison to a team-based service model when engaging and supporting patrons. The previous liaison model fostered relationships and met the needs of patrons for many years. With the new team-based model, the team strives to mirror the collaboration and interdisciplinary approach embraced by clinicians and health care professionals at Yale New Haven Hospital (YNHH). This approach allows librarians to lean into their strengths and interests, reduces workload, mitigates burnout, and provides a space for collaboration and feedback. This presentation will explore the advantages and challenges of transitioning to a new model, highlight similar initiatives at peer institutions, and demonstrate how this approach supports both patrons and librarians in innovative ways. The unique perspective of being connected to a health system and a university library will also be examined. Attendees will leave with practical takeaways and resources to consider implementing in their own workplaces.

# Abstract: LT4. La traduction et l'adaptation des RELs pour combler le manque de ressources bilingues

Peter Farrell, Nigèle Langlois

Université d'Ottawa

Créer des ressources pédagogiques à partir de rien est souvent une tâche ardue, surtout lorsqu'il faut les produire en français et en anglais. Les ressources pédagogiques en français pour soutenir la synthèse des connaissances sont encore peu nombreuses. Cependant, la traduction et l'adaptation des supports existants offrent un moyen pratique de faciliter leur élaboration et de combler cette lacune. La Bibliothèque des sciences de la santé de l'Université d'Ottawa s'est récemment lancée dans une telle initiative. Nos bibliothécaires de recherche ont fréquemment partagé le guide « Searching the Literature: A Guide to Comprehensive Searching in the Health Sciences », élaboré par l'Université de Toronto. Cependant, nos collègues travaillant dans des environnements francophones ont régulièrement souligné le manque de ressources équivalentes en français. En réponse à cela, en accèdent à une bourse REL, nous avons entrepris la traduction et l'adaptation complètes de ce guide. Dans cette présentation éclair, nous vous présenterons le déroulement du projet, les défis que nous avons rencontrés et nos objectifs pour l'avenir. Nous espérons vous inciter à envisager la traduction et l'adaptation des ressources sur lesquelles vous vous appuyez, et à les partager avec vos collègues d'autres institutions francophones.

# Abstract: LT5. Locating and analyzing content in LibGuides geared towards physician assistant students

Karly Gunson

University of Saskatchewan

**Introduction:** While the physician assistant (PA) role is not new in North America, there has been a proliferation of second entry and graduate-level academic programs within the past 10 years. Three out of six Canadian programs have begun in the five years alone. Providing library services to clinical students is central to evidence-based practice and fostering research confidence. Librarians may provide support through research and subject LibGuides (https://www.springshare.com/libguides). There are several studies analyzing LibGuides for other academic programs, yet few for PA studies. Indeed, there have only been three articles published by librarians investigating PA-specific information services in the past eight years, none of which are in the Canadian context. There is a need to scan what information is available to Canadian PA students so they can be better supported. **Methods:** This analysis involved searching for Canadian university PA program-specific LibGuides to locate and report program specific content. Data was categorized and descriptive analysis conducted. **Results:** Among Canadian university PA LibGuides, there is considerable overlap in content. Most guides share information on general medical sciences/foundations, access to textbooks, research support and literature review help, and clerkship. Some guides have sections for professional competencies and transition into practice (with exam and certification information). **Discussion:** There are limited librarian-led investigations on PA-specific library content, programming, and services. Through identifying and analyzing LibGuide content that is currently available to Canadian PA students, we can pinpoint information gaps and address student needs. Further, in documenting what content is available today, we can have a baseline for future comparisons.

# Abstract: LT6. Concerning past: confronting anatomy texts with Nazi authors

Amy Rutherford

Queen's University

**Introduction:** Recently, librarians at Bracken Health Sciences Library realized that the library held anatomy books written and illustrated by members of the Nazi party. This lightning talk will describe the history of these books by Pernkopf and Spalteholz, and the decision-making process that led to moving them to W.D. Jordan Rare Books and Special Collections. **Description:** Curator, Brendan Edwards and Librarian, Amy Rutherford met to assess two sets of anatomy books. The Curator's criteria for acceptance included condition, publication year, edition, and original binding. Although the Spalteholz set was incomplete, the condition and original binding were sufficient for relocation. The illustrators of these texts used holocaust victims as models for their illustrations; thus, the decision was that they should be removed from regular circulation. **Outcomes:** A total of three volumes were removed from Bracken. A public note will be added to the MARC records, which will be viewable to library users who may encounter these works. It is critical that a public note explains the problematic history of these texts and why they were removed from circulation. These books can still be viewed or studied upon request at W.D Jordan Rare Books and Special Collections. **Discussion:** This presentation will hopefully spark curiosity about these anatomy books, and whether there are copies circulating at other Canadian health sciences libraries. It is important that these volumes not be eradicated, to avoid the erasure of historical evidence of the holocaust and works that have a racist or xenophobic past.

# Abstract: LT7. What you see depends on where you sit: part deux

Chris Cooper^1^, Zahra Premji^2^

^1^University of Bristol, ^2^University of Victoria

**Introduction:** In 2021, we reported a proof-of-concept study which demonstrated that geographical location (where you sat) affected Google search results (what you see). Using a Virtual Private Network (VPN) to mimic the effect of geographic location, we were able to prove that a person based in Australia would see different Google search results to a person based in America. Now we consider if using a VPN might be an effect modifier compared to searches conducted by humans, physically in those locations. **Methods:** We sampled five countries using the same search string. We compared the search results retrieved using a VPN concurrently with a live search conducted by a human at each location. **Discussion/Conclusion:** There is little guidance on the method of websearching, despite it being a recommended supplementary search method for systematic reviews. This study extends our previous work and will show if the use of VPN is reliable or not compared to a human searcher in the country of interest.

# Abstract: LT8. A new chapter: the formation of the Atlantic Health Libraries Association / Association des bibliothèques de la santé de l'Atlantique

Jordan Pike

Memorial University

**Introduction:** In 2024, the Maritimes Health Libraries Association / Association des bibliothèques de la santé des Maritimes (MHLA/ABSM) and the Newfoundland and Labrador Health Libraries Association (NLHLA) merged to form the Atlantic Health Libraries Association / Association des bibliothèques de la santé de l’Atlantique (AHLA/ABSA). This lightning talk outlines the motivations, process, and outcomes of the merger, offering insights for other associations considering similar transitions, and serves as an introduction to the new CHLA/ABSC chapter. **Description:** The merger was driven by declining membership, the normalization of virtual meetings post-COVID-19, and a desire for increased engagement. After a trial collaboration and membership votes, working groups addressed scope of practice, financial integration, executive structure, and governance. **Outcomes:** Key milestones included ratifying a new constitution and by-laws, electing an executive, and adopting a new name. Rebranding efforts focused on consolidating communication platforms and developing a logo through a two-phase design process. Practical steps such as merging bank accounts and refining governance structures highlighted the complexities of integration. **Conclusion:** By building on the legacy of its predecessor associations and adapting to current needs, AHLA/ABSA is positioning itself for future success in serving health library professionals across the Atlantic Provinces.

# Abstract: LT9. Opening doors: a new chapter in medical librarianship

Victoria James

Toronto Metropolitan University's Bikram S. Dhillon and Family Medical Library

The launch of the Bikram S. Dhillon and Family Medical Library at Toronto Metropolitan University marks a significant moment in Canadian health librarianship. Opening its doors in September 2025, the library supports the new School of Medicine with a focus on learner-centered, equity-informed service. This lightning talk reflects on the early months of operation and the ongoing work of building a library that listens, adapts, and grows alongside its community. As CHLA/ABSC celebrates 50 years of health expertise, this presentation considers what it means to build something new in a field shaped by longstanding practice. We will share insights from the library's foundational phase, including approaches to instructional design, outreach, and resource development that responds to the evolving needs in medical education. The talk will also highlight how librarianship continues to adapt, bridging established practice with innovation and evidence with empathy. As the library opens in phases, this session offers a snapshot of a library in motion: shaped by collaboration, learner responsiveness, and inclusive library practice. We invite attendees to consider how new spaces can reflect enduring commitments while making room for change. This submission is intended to spark conversation about the future of health libraries and to honour the collective work of those who have built and continue to build spaces for learning, care, and connection.

# Abstract: LT10. Modern time capsule: honouring leadership experience to shape future health sciences library leaders

Angela Murrell^1^, Sunny Chung^2^, Kay Strahan^3^

^1^University of Arizona, ^2^Stony Brook University, ^3^University of Arkansas for Medical Sciences Northwest Regional Campus

**Introduction:** Health sciences librarians are currently seeing a shift in the workforce as many leaders are approaching retirement, leaving leadership positions open for professional organizations to restructure and fill with new leaders educated with specific leadership offerings. This study aims to investigate pressing needs for leaders in the profession and how those needs will shape future leaders in national and international organizations of health sciences librarianship. **Methods:** This study used a mixed-methods design to quantitatively and qualitatively analyze the attitudes and perceptions of leaders in the profession regarding opportunities for leadership development and their current needs and priorities. Researchers designed and piloted a survey on Qualtrics to identify pressing leadership needs and how professional organizations can develop better educational offerings to support these needs. The survey invitation and link were distributed to relevant health sciences listservs in North America. The qualitative analysis developed key themes utilizing Taguette. **Results:** Among 157 survey respondents, most were academic health sciences librarians (n=87), followed by hospital librarians (n=51), with fewer from other settings. Senior leaders were underrepresented, suggesting turnover or survey reach limitations. Qualitative analysis revealed inconsistent mentorship, program suitability, and insufficient topic depth. The most important leadership skills centered around advocacy, management, and strong leadership vision. **Discussion:** Findings suggest a growing need for cost-effective and targeted training for leadership in health sciences libraries. Particularly in accessibility, relevance, and mentorship opportunities, ensuring educational programs better equip librarians for leadership roles. Recommendations will aim to align training with participant needs and foster a more robust leadership pipeline.

# Abstract: LT11. Because grey still matters: advancing grey literature search approaches at Canada's Drug Agency

Sharon Bailey, Robin Featherstone, David Kaunelis

Canada's Drug Agency

**Introduction:** In 2022, Research Information Specialists from CADTH (now Canada’s Drug Agency) presented at the CHLA-ABSC conference on modernizing our bibliographic and grey literature search tools. To build on this work and address the evolving information needs of Canada’s Drug Agency, Research Information Services investigated the current state of our grey literature search approaches and made recommendations for enhancement in 2024. In 2025, we began implementing these recommendations. **Methods:** We sought input from our Research Information Specialists about the value and efficacy of our current grey literature search approaches and tools through an internal focus group and survey. We also reached out to the HTA community on the visibility and usefulness of Grey Matters, our public-facing grey literature checklist tool, via a survey on the Health Technology Assessment International Information Retrieval Group email listserv. **Results:** Based on internal and external feedback, our recommendations for grey literature searching processes focus on 4 areas: (1) project initiation and consultation, (2) enhancements to our internal grey literature checklists, (3) reporting and internal documentation of grey literature search approaches, and (4) increasing awareness of Grey Matters. Project work to implement recommendations began in 2025 with delivering enhancements to our grey literature checklists and updating their maintenance processes. **Discussion:** The findings from our investigation informed recommendations to revitalize and enhance our grey literature search approaches and tools. We are currently implementing these recommendations and will repeat this evaluation within 5 years to ensure sustained viability of our grey literature search methods.

# Abstract: LT12. Clinicaltrials.embase?

Zahra Premji^1^, Chris Cooper^2^

^1^University of Victoria, ^2^University of Bristol

**Introduction:** The Cochrane Handbook recommends searching clinical trials registries for systematic reviews of interventions, including specifically clinicaltrials.gov (CTG). Trial records from CTG are also indexed in the World Health Organization International Clinical Trials Registry Platform (ICTRP), Cochrane Central and, as of October 2025, in the Embase database. Current guidance recommends searching CTG through the native platform as well as the ICTRP platform, due to differences in search functionality and indexing of the records. With Embase now including CTG records, we sought to determine whether all relevant CTG records can be retrieved from searching Embase. **Methods:** We conducted an exploratory information retrieval study on the recall of relevant CTG records within an Embase search. We used search strategies and included studies from previously published Cochrane reviews of interventions, published in 2025. Cochrane reviews that searched Embase, CTG, and ICTRP were eligible for inclusion. We replicated their searches in each source, and exported the records. We then checked each retrieval set against all CTG trial records/numbers mentioned in the ‘references to studies included in this review’ section of each review, and calculated recall for each source. For missed CTG records, we confirmed whether it was present in the platform. And in the case of Embase, we explored why some were missed from the search. **Discussion/Conclusion:** The findings of this study provides initial evidence on whether CTG records can be reasonably retrieved through a search of Embase, and whether the existing evidence stating that both CTG and ICTRP should be searched separately is still true.

# Abstract: LT13. Votre mission si vous l'acceptez : intégrer la bibliothèque !

Diana Maude Couture, Julie Desautels

CISSS de Chaudière-Appalaches

**Contexte :** Dans le cadre du développement continu des services informationnels en milieu hospitalier, la Bibliothèque du CISSS de Chaudière-Appalaches a été intégrée au processus d’analyse de projets du bureau de projet de l’établissement. Cette initiative vise à reconnaître l’expertise documentaire comme un levier stratégique dans la planification, la mise en oeuvre et l’évaluation des projets cliniques et administratifs. **Objectif :** Renforcer la qualité et l’efficacité de la prise de décision en intégrant les bibliothécaires et techniciens en documentation dès les premières étapes du cycle de vie des projets, notamment par la recherche d’informations probantes, la veille stratégique et la gestion des connaissances. **Méthodes :** Les bibliothécaires participent désormais, lorsque jugé pertinent, aux comités du bureau de projets, à l'analyse sommaire des données, à l'identification des sources fiables, aux décisions liées aux indicateurs de réussite, ainsi qu'à la diffusion et conservation des résultats. Une correspondance régulière par courriel entre les bibliothécaires et le bureau de projet permet de maintenir le lien et d’éviter de se perdre de vue. **Résultats :** L’intégration a permis une meilleure cohérence des informations utilisées dans les projets, une réduction du temps de recherche, et une amélioration de la qualité des analyses. **Conclusion :** L’inclusion de la Bibliothèque dans le processus d’analyse de projet démontre la valeur ajoutée des professionnels de l’information en milieu hospitalier. Ce modèle de collaboration favorise une culture organisationnelle fondée sur les données probantes et peut être adapté à d’autres contextes institutionnels.

# Abstract: LT14. Case report of a challenging search - interlibrary collaboration supporting research on patients without doctors

Sabine Calleja^1^, Alexandre Amar-Zifkin^2^

^1^McGill University, ^2^Université de Montréal

**Introduction:** Literature searching is arguably a health science librarian's bread and butter. Experienced librarians develop intuitions drawn from their searches, and subject matter knowledge accumulates from reference interviews and background readings. Collaboration with librarian colleagues, both internal and external, invites new points of view, draws on different bodies of knowledge and experience, and helps break down naturally-occurring knowledge silos. However, both of these processes - engaging with literature and searches to develop wider-ranging knowledge, and outreach to colleagues - are often personal, private, and specific to the individual librarian. **Description:** Drawing on recent publications proposing that health science librarians write up case reports of challenging searches, we present a particularly tricky search and the unexpected flashes of inspiration and insight that resulted in the user receiving useful articles from their librarian. **Discussion:** An increasing proportion of patients in Quebec, along the entire continuum of care, do not have access to a family doctor or GP (general practitioner). We describe a research project seeking to explore models of care for cancer survivors without a GP, and more broadly the particularities of searching for articles about people who do not have a doctor. We will present what seem to be the most appropriate and useful MeSH terms, as well as alternative conceptualizations of the search that added to the pool of pertinent articles retrieved; the products of an informal and fruitful collaboration across two institutions.

# Abstract: LT15. Into the meta-search: a case report of capturing information retrieval methods

Melissa Walter, Robin Featherstone, David Kaunelis

Canada's Drug Agency

**Introduction:** As part of a comparative analysis on automation and AI tools for information retrieval by Canada’s Drug Agency, we conducted a focused literature review on how three tools (SpiderCite, Lens.org, and Microsoft Copilot) had been used in evidence synthesis. The literature review required adapting our standard search approach to capture reports based on information retrieval methods reported in the full-text. **Methods:** We designed strategies in PubMed Central (PMC), Google Scholar, and Europe PMC to target uses of the three tools in evidence reviews. Given functionality differences with these three databases, as well as specific challenges related to the search terms for the Lens.org and Copilot concepts, we adopted different approaches for each search. **Results:** In PMC and Europe PMC, we used search functions to capture keywords within the methods sections of review articles. We also used PMC’s search field for the body of the text, not including references or abstracts. In-depth searching of Google Scholar confirmed that syntax does not function correctly when used according to Google guidance, and that metadata of search results is messy and requires significant reformatting. However, tools such as Publish or Perish significantly eased the workload of working with Google Scholar. **Discussion:** The approaches used in this case to search non-standard fields in full-text databases and to utilize a records management tool with Google Scholar significantly focused results and reduced the screening burden for methods research. We will employ the approaches established from the literature review from this case for future methodological research at Canada’s Drug Agency.

# Abstract: LT16. Another tool in the toolbox or a tipping point? Librarians and generative AI for search strategy development

Zahra Premji^1^, Kaitlin Fuller^2^, Erica Nekolaichuk^3^

^1^University of Victoria, ^2^St. Francis Xavier University, ^3^University of Toronto

**Introduction:** In 2025, the creation of the RAISE (Responsible Use of Artificial Intelligence in Evidence Synthesis) guidelines signaled increased interest in exploring ethical use of artificial intelligence tools for improving sustainability in knowledge synthesis (KS) work. Over the last 3 years, there has been an increase in the development and availability of generative AI tools, including chatbots, AI academic search engines, and AI add-ons/assistants connected to traditional databases. As librarians who support KS, we were interested in finding out whether health librarians were using generative-AI tools in their KS practice, and if so, when and how. **Methods:** We used a cross-sectional survey for our study. Eligibility was restricted to librarians currently supporting at least one health sciences program, and involved in knowledge synthesis as a consultant, collaborator, or both. The survey included questions related to demographics and librarians' use of generative AI tools in the context of their roles as educational consultants and collaborators.

# Abstract: LT17. Demonstrating responsible use of artificial intelligence through creation and curation of closed system chat bot agents

Margaret A. Hoogland

University of Toledo

**Introduction:** During Fall 2025 sessions with first-year medical students, the Clinical Medical Librarian (CML) started discussing ways Artificial Intelligence (AI) could be used to enhance search strategies, more quickly retrieve instruction to author information for journals, and so forth. CML recognized that sharing articles about proper ways to use and cite AI is one step – but it is not sufficient to address faculty, staff, and learners’ interest in and exploration of AI. **Description:** By developing closed system chat bot agents, CML can improve accessibility of existing information on Scholarly Activities, Journal Metrics, and provide some basic definitions for commonly asked questions by faculty and learners about selecting journals, critical appraisal, predatory journals, and indexing. The Chat Bot Agents will include a way for users to get assistance, if they are in a crisis. **Outcomes:** CML will provide a structure so that a patron’s prompt will retrieve information from existing sources curated by CML. The Chat Bot Agents will give patrons, who are interested in AI, an opportunity to more easily access the information, while also practicing and improving how they write prompts. **Discussion:** By developing and maintaining these closed system Chat Bot Agents, CML demonstrates the importance of creating a good research question (or prompt) before embarking on a research project.

# Abstract: LT18. Keeping current on AI tools for evidence synthesis: an example using Elicit Systematic Review

Megan Kennedy, Liz Dennett, Janice Kung, Lisa Tjosvold, Connie Winther

University of Alberta

Keeping current on emerging AI tools for evidence synthesis can be a challenge for librarians who support knowledge synthesis projects like systematic, scoping, and other types of reviews. These tools are rapidly evolving and are increasingly being integrated into users' review processes. However, many AI tools for evidence synthesis exist behind paywalls with limited free trials and opportunities for librarians to fully explore all the features offered by the tool. This can greatly limit librarians' knowledge about how they might be used by a research team conducting a knowledge synthesis project. This presentation is a case study that will showcase an approach that was employed by health sciences librarians at the University of Alberta for exploring and learning about the AI tool, Elicit Systematic Review. The process used was an informal one but still provided promising insights into the strengths and limitations of this AI tool. Librarians were able to develop evidence-based recommendations to provide practical guidance to researchers who are using this tool.
